# A phase-I trial of pre‐operative, margin intensive, stereotactic body radiation therapy for pancreatic cancer: the ‘SPARC’ trial protocol

**DOI:** 10.1186/s12885-016-2765-4

**Published:** 2016-09-13

**Authors:** Daniel L. P. Holyoake, Elizabeth Ward, Derek Grose, David McIntosh, David Sebag-Montefiore, Ganesh Radhakrishna, Neel Patel, Michael Silva, Somnath Mukherjee, Victoria Y. Strauss, Lang’o Odondi, Emmanouil Fokas, Alan Melcher, Maria A. Hawkins

**Affiliations:** 1 CRUK/MRC Oxford Institute for Radiation Oncology, Department of Oncology, University of Oxford, Old Road Campus Research Building, Roosevelt Drive, Oxford, OX3 7DQ UK; 2 Oncology Clinical Trials Office, Department of Oncology, University of Oxford, Old Road Campus Research Building, Roosevelt Drive, Oxford, OX3 7DQ UK; 3 The Beatson West of Scotland Cancer Centre, 1053 Great Western Rd, Glasgow, G12 0YN UK; 4 The University of Leeds, Cancer Research UK Leeds Centre,14 Leeds Institute of Cancer and Pathology, Cancer Genetics Building, St James’s University Hospital, 15 Beckett Street, Leeds, West Yorkshire LS9 7TF UK; 5 Leeds Cancer Centre, Leeds Teaching Hospitals NHS Trust, Bexley Wing, Beckett Street, Leeds, LS9 7TF UK; 6 Oxford University Hospitals NHS Foundation Trust, Old Road, Headington, Oxford, OX3 7LE UK; 7 Centre for Statistics in Medicine, Nuffield Department of Orthopaedics, Rheumatology and Musculoskeletal Sciences, University of Oxford, Botnar Research Centre, Windmill Road, Oxford, OX3 7LD UK; 8 The Institute of Cancer Research, Chester Beatty Laboratories, 237, Fulham Rd, London, SW3 6JB UK

**Keywords:** Pancreatic cancer, Borderline-resectable, Stereotactic body radiation therapy (SBRT)

## Abstract

**Background:**

Standard therapy for borderline-resectable pancreatic cancer in the UK is surgery with adjuvant chemotherapy, but rates of resection with clear margins are unsatisfactory and overall survival remains poor. Meta-analysis of single-arm studies shows the potential of neo-adjuvant chemo-radiotherapy but the relative radio-resistance of pancreatic cancer means the efficacy of conventional dose schedules is limited. Stereotactic radiotherapy achieves sufficient accuracy and precision to enable pre-operative margin-intensive dose escalation with the goal of increasing rates of clear resection margins and local disease control.

**Methods/Design:**

SPARC is a “rolling-six” design single-arm study to establish the maximum tolerated dose for margin-intensive stereotactic radiotherapy before resection of pancreatic cancer at high risk of positive resection margins. Eligible patients will have histologically or cytologically proven pancreatic cancer defined as borderline-resectable per National Comprehensive Cancer Network criteria or operable tumour in contact with vessels increasing the risk of positive margin. Up to 24 patients will be recruited from up to 5 treating centres and a ‘rolling-six’ design is utilised to minimise delays and facilitate ongoing recruitment during dose-escalation. Radiotherapy will be delivered in 5 daily fractions and surgery, if appropriate, will take place 5–6 weeks after radiotherapy. The margin-intense radiotherapy concept includes a systematic method to define the target volume for a simultaneous integrated boost in the region of tumour-vessel infiltration, and up to 4 radiotherapy dose levels will be investigated. Maximum tolerated dose is defined as the highest dose at which no more than 1 of 6 patients or 0 of 3 patients experience a dose limiting toxicity. Secondary endpoints include resection rate, resection margin status, response rate, overall survival and progression free survival at 12 and 24 months. Translational work will involve exploratory analyses of the cytological and humoral immunological responses to stereotactic radiotherapy in pancreatic cancer. Radiotherapy quality assurance of target definition and radiotherapy planning is enforced with pre-trial test cases and on-trial review. Recruitment began in April 2015.

**Discussion:**

This prospective multi-centre study aims to establish the maximum tolerated dose of pre-operative margin-intensified stereotactic radiotherapy in pancreatic cancer at high risk of positive resection margins with a view to subsequent definitive comparison with other neoadjuvant treatment options.

**Trial registration:**

ISRCTN14138956. Funded by CRUK

## Background

Pancreatic cancer is responsible for only 3 % of cancer diagnoses but 5 % of cancer deaths, making it the fifth leading cause of cancer death in the UK (9408 cases in 2013 and 8817 deaths in 2014) [1]. Radical surgical resection represents the only chance of long-term disease control, but was possible for just 8 % of UK patients diagnosed in 2006–2010 [2]. In Europe standard practice for resectable disease is surgery followed by chemotherapy, but for these patients median survival is only 24 months and 50 % of patients will suffer local recurrence [3] suggesting that further improvement in multi‐modal therapy is required.

The failure to consistently achieve microscopic surgical clearance contributes to the high rates of disease relapse: alongside tumour size, grade and lymph node metastases, resection margin involvement has been repeatedly shown to predict long-term survival [4–6]. Accurate margin assessment requires comprehensive examination of the surgical specimen and rates of margin involvement are higher when a standardised protocol is used [7]. Despite centralization of services and improvement in diagnostic radiology, positive margins were reported in >35 % of patients in the largest multi‐national adjuvant trial in pancreatic cancer involving over 1000 patients (ESPAC 3), which again reinforced that patients with positive margins have poor outcome (hazard ratio 1.35, 95 % confidence interval 1.17‐1.56, *p* < 0.001) [8].

A small proportion of patients present with pancreatic tumours that can be classified as “borderline-resectable”, defined by a limited extent of vascular invasion. For these patients resection is possible but is likely to require vascular reconstruction and there is an especially high risk of positive resection margins, reported to be 62.9 % in the UK [9]. The concept of Borderline Resectable Pancreatic Cancer (BRPC) was initially described in 2001 by Mehta et al., describing 15 cases initially assessed as “marginally resectable” but where resectability was improved with preoperative chemo-radiotherapy [10]. The definition of BRPC was refined by an AHPBA/SSO/SSAT consensus statement [11, 12], now adopted by the NCCN [13]:-

- Venous involvement of the SMV or PV with distortion or narrowing of the vein or occlusion of the vein with suitable vessel proximal and distal, allowing safe resection and reconstruction
- Gastro‐duodenal artery encasement up to hepatic artery with either short segment encasement or direct abutment of the hepatic artery, without extension to the celiac axis
- Tumour abutment of the SMA not to exceed greater than 180° of the circumference of the vessel wall.

An alternative definition of BRPC by the MDACC has not been as widely recognised [14]. A multidisciplinary team approach is needed to assess the likelihood of attaining negative margins and this should remain the key consideration in determining if a patient is a potential candidate for resection [14, 15].

The management of BPRC is controversial, and there is increased interest in pre-operative treatment strategies that may improve outcomes [16]. Theoretical advantages include a greater chance of delivering full dose treatment, while drawbacks include possible over-treatment of resectable disease, the need for a biopsy and stent to relieve obstruction while awaiting surgery. Many patients presenting with localised disease develop frank metastases shortly after diagnosis and if this occurs during induction therapy these patients can be spared inappropriate and futile surgery [14].

Pre-operative (chemo) radiotherapy aims to treat the tumour in situ to achieve tumour regression and facilitate curative resection. This approach has become standard of care for some tumour sites, having been shown to reduce local recurrence in rectal cancer [17], and increase overall survival in oesophageal cancer [18], but is not established in pancreatic cancer due to limited effectiveness of standard dose schedules, despite potential advantages over postoperative treatment [19]. A complete tumour response is only seen in around 5 % of patients [20], though a larger proportion of patients (35 %) benefit from tumour down-staging to achieve resectability [21] and patients who successfully complete neoadjuvant chemoradiotherapy and go on to have resection tend to have high rates of clear resection margins [22, 23] and reduced rates of local recurrence [24, 25].

Stereotactic body radiation therapy (SBRT) combines rigorous immobilisation and image-guidance with hypofractionation, to reduce radiotherapy planning margins and treat small targeted volumes with greater accuracy and precision, supporting safe delivery of much higher biologically effective doses. It has been shown to be a highly-effective local treatment option in unresectable pancreatic cancer with high rates of local control achieved using treatment delivered in a single exposure [26–29], and when treatment was delivered in 3–5 fractions lower rates of toxicity were seen without compromising disease control [30–35].

The specific goal in treating BRPC with pre-operative radiotherapy will be improve the chances of achieving clear resection margins, reduce risk of local recurrence and achieve chances of long-term survival similar to those for patients with initially resectable tumours. Neoadjuvant treatment has been shown to be deliverable and effective in BRPC, such as in a series of 160 patients described by Katz et al., among whom 78 % completed preoperative therapy and restaging, and 41 % underwent pancreatectomy, with a 94 % rate of clear margins [36].

The premise of the SPARC trial is a Margin-Intense radiotherapy concept where the whole tumour is treated to a minimum dose while a simultaneous integrated boost (SIB) is used to deliver additional dose to the margin of tumour around structures such as the superior mesenteric artery (SMA), SMV or portal vein and retroperitoneal margin. This approach complements surgery as these are the most difficult margins to resect and are at highest risk of positive surgical margins. Prospective dose-escalation aims to establish the maximum tolerated dose for this treatment paradigm for subsequent definitive investigation of efficacy. Once the maximum tolerated dose (MTD) is established this will provide a platform to integrate with optimal systemic treatment.

## Methods/Design

### Study design

The SPARC trial is a phase I dose-escalation study using the rolling six design [37] to establish MTD of SBRT delivered in the pre-operative setting for borderline resectable pancreatic cancers. This method minimises delays in the dose escalation phase [37]. Patients will complete SBRT in approximately one week and surgery will take place 5–6 weeks after radiotherapy. Patients will be on the study for 36 weeks from registration to end of treatment visit followed by standard care for two years post SBRT day one for the last patient, or when all participants have died.

The trial opened in April 2015 and will recruit up to 24 patients from five UK centres (Oxford, Glasgow, Leeds, Newcastle and Nottingham). The SPARC trial is conducted in accordance with the Helsinki Declaration (1996) and the regulatory requirements for clinical trials of an investigational medicinal product under the European Union Clinical Trials Directive. The trial is approved by the National Research Ethics Service Committee South Central – Oxford B (REC reference: 15/SC/0059) and is sponsored by the University of Oxford, with funding from Cancer Research UK. Data submission for the SPARC trial is via electronic submission into the online database system OpenClinica by site staff. The Oncology Clinical Trial Office (OCTO) coordinates and manages the trial while the Centre for Statistics in Medicine (University of Oxford) will perform all statistical analysis.

### Participants

The inclusion and exclusion criteria for the SPARC trial are summarised in Table 1.

**Table 1:** SPARC Inclusion/Exclusion criteria

Inclusion criteria	Exclusion criteria
1. Borderline resectable localised tumour of the pancreatic head/uncinate process/body as per NCCN Guidelines (tumours of the tail of pancreas are not eligible for inclusion) or operable tumour in contact with vessels increasing the risk of positive margin as defined by CT +/− MRI +/− PET criteria within 28 +/− 7 days prior to trial entry, de novo or following systemic treatment. 2. Histologically proven pancreatic ductal adenocarcinoma or cytological proven pancreatic malignancy 3. Able to undergo biliary drainage using a stent 4. Deemed fit and suitable for surgical resection. 5. No overt metastases or uncertain status with investigations suspicious of possible metastatic disease (e.g., small equivocal pulmonary nodule(s)). 6. Male or female, Age ≥16 years 7. Life expectancy of at least 6 months 8. ECOG performance status 0–1 9. The patient is willing and able to comply with the protocol for the duration of the study, and scheduled follow-up visits and examinations 10. Written (signed and dated) informed consent and be capable of co-operating with protocol 11. Haematological and biochemical indices within specified ranges.	1. Definitive metastatic disease or local disease that cannot be encompassed in the SBRT field. 2. History of previous or concurrent malignancy diagnoses for which the expected prognosis is likely to be worse than that of the current diagnosis of pancreatic cancer (excludes for example: e.g., localised prostate cancer, early colorectal cancer, early breast cancer, curatively-treated basal cell carcinoma of skin, carcinoma in situ of cervix; curatively treated cancer of other sites who are recurrence free for >3 years). 3. Serious medical or psychological condition precluding trial intervention. 4. Previous upper abdominal or right chest wall radiotherapy where 30 % of the liver has received >15Gy. 5. Pregnancy: Pregnant or breast-feeding women are ineligible. Women of childbearing potential must use effective methods of contraception. 6. Any other psychological, social or medical condition, physical examination finding or laboratory abnormality that the Investigator considers makes the patient a poor trial candidate or could interfere with protocol compliance or the interpretation of the trial results.

### Interventions

At registration patients are assigned to the appropriate SBRT dose level (Table 2) and will receive 5 fractions of SBRT over 5–8 days. The dose schedules selected have been designed to achieve approximately equivalent dose to the tumour to that used in conventionally fractionated radical radiotherapy (50-66Gy in 2Gy fractions) while the dose selected for the ‘at risk volume’ (area at risk of positive margin) is higher, to achieve increased likelihood of ablation of tumour cells. A maximum of 4 dose levels (including one de-escalated dose level) are expected to be needed to establish the MTD.

**Table 2:** Radiotherapy dose escalation levels

Radiotherapy dose level	Tumour (PTV)				Area at risk of R1 (PTV_R)			
	Dose/# [Gy]	Total dose [Gy]	*BED [Gy_10_]	EQD_2_ [Gy]	Dose/# [Gy]	Total dose [Gy]	*BED [Gy_10_]	EQD_2_ [Gy]
Level −1	6	30	50	40	8	40	72	60
Level 1	6	30	50	40	9	45	88	71.5
Level 2	6.5	32.5	56	45	9.5	47.5	92	77.2
Level 3	7	35	62	50	10	50	100	83.3

*BED [Gy_*10*_ *]* biologically effective dose for acute reacting tissues (α/β = 10), *EQD*_*2*_ equivalent dose in 2-Gy fractions

Dose level assignment will be based on the number of dose limiting toxicities (DLTs) observed, and the number of patients have no DLTs yet, but are still in the DLT assessment period as defined in the outcome measure below (i.e., toxicity data pending/evaluable not yet). In this trial design, the first two patients are treated at level 1. The third patient will be treated at level −1 if two DLTs have been observed. If at least one patients enrolled is not evaluable yet and less than 2 DLTs have been observed, this third patient will be entered at the same dose level, Level 1. For the fourth patient, if toxicity data is pending for at least one of the patients enrolled, the fourth patient will be allocated to the same dose level. But, if there are two or more DLTs when the fourth patient enters the study, the dose level will be de-escalated. The dose level will be escalated if there is no DLT after the first three patients finish the DLT assessment period. This process will be repeated for patients five and six. After the sixth patient is enrolled to a dose cohort, accrual will be only temporarily suspended until toxicity data is fully available for these six patients and/or a decision from the Trial Management Group (TMG) is made. Recruitment will stop once the MTD is identified.

### Outcome measures and definitions

The primary endpoint of the SPARC study is MTD, defined as the highest level of SBRT at which no more than 1 of 6 patients or 0 of 3 patients experiences a DLT in the period from starting SBRT to one month post-surgery or one month after the evaluation in week 3 post-SBRT if no surgery takes place. DLT is defined as these possible adverse events (AEs) (according to CTCAE v4.03), seen in the DLT assessment period:

1. ≥ Grade 3 upper gastro-intestinal (GI) bleeding
2. ≥ Grade 4 nausea/vomiting uncontrolled after 48 h of standard treatment
3. ≥ Grade 4 pancreatitis not stent related
4. Interruption of SBRT >1 week due to SBRT-related AEs
5. ≥ Grade 4 vascular events: SMV thrombosis, bowel ischaemia due to SMA arteritis/stenosis, friable vessels at surgery
6. Other AEs that the TMG agrees to be dose limiting and possibly related to SBRT such as ≥ grade 3 gastrointestinal fistula >30 days after surgery

Secondary outcomes include resection rate, resection margin status rates, rate of pathological complete response, late SBRT toxicity, efficacy and long term safety. Resection rate is defined as the proportion of patients with radical oncological resection of pancreatic cancer over the total enrolled patients. Resection margin status (R0/R1/R2) is defined according to the standards of the Royal College of Pathologists, and response rate is defined as the rate of patients with pathological complete response over the total enrolled patients. Late toxicity is defined as any GI AE/other AE > grade 2 occurring between 1 month and 6 month post-surgery. Additional secondary outcomes include efficacy and long term safety as indicated by progression free survival (PFS) and overall survival (OS). PFS is defined as the time from date of first SBRT dose to the first date of progression or death for any cause, or is censored at the date of last known follow-up date if they are not observed to die during the course of the trial. OS is defined as the time from date of first SBRT dose to the date of death for any cause or is censored at the last known follow-up date. Exploratory outcomes include relationships between imaging and pathology (treatment response and resection margin status) and changes in markers of the innate and adaptive immune response before and during SBRT (cytological assessments and levels of interferon-related RNA).

### Sample size considerations

Patient numbers will be determined by the primary endpoint of establishing the MTD, which will require a maximum of 24 evaluable patients. An evaluable patient for primary endpoint analysis is defined as one who has received at least one fraction of SBRT and experiences a DLT, or a patient who has received at least four fractions of SBRT and is assessed one month post-surgery (or assessed at similar time for DLT if no surgery). Patients not evaluable for primary endpoint will be replaced, but a patient who does not become evaluable for the primary endpoint may continue to be followed up for surgical outcome, PFS and OS as appropriate, unless consent is withdrawn.

### Statistical analysis

All analyses will follow a statistical analysis plan written in accordance with current standard operating procedures. Baseline characteristics will be summarised (number/frequency) for all enrolled patients. Patients who died or withdrew before treatment started or fail to complete the required safety observations will be described separately. Primary MTD analysis will concern the frequency of patients with no DLT according to dose-escalation of SBRT. Descriptive statistics will summarise the DLT’s and safety variables with patients grouped according to dose level received. Secondary outcomes (resection rate, resection margin status and response rate) associated with SBRT will be summarised by descriptive statistics along with numbers and percentages. Late toxicity will be summarised by descriptive statistics for all patients who received at least one dose of SBRT treatment. Physical exam, haematology and biochemistry data will be summarised across time. Both PFS and OS estimates at one and two years and 90 % CI will be provided together with median estimates. A Kaplan-Meier curve will be plotted with 90 % CI. A trial summary will record patient recruitment and trial decision-making.

### Monitoring committees

The Radiotherapy and Imaging Trials Oversight Committee (RIOC) is the independent committee for SPARC covering both data management committee and oversight responsibilities as delegated by the Sponsor and will meet at least once annually to review and ratify substantial decisions.

## Discussion

This study builds on the current evidence base in SBRT for pancreatic cancer, which has so far been largely used in the locally advanced setting with promising results and aims to take it a step further by prospectively evaluating the safety and benefit of pre‐operative margin-intense SBRT.

A five-fraction schedule aims to optimise the balance of short duration and tumouricidal biologically-effective dose to the tumour with acceptable risk of late normal tissue injury. Potential clinical benefits of a significantly hypofractionated radiotherapy regimen compared with a protracted conventional 6-week course of chemoradiotherapy include a shorter enforced delay to definitive surgery or alternatively more straightforward integration with systemic therapy regimens.

**Fig. 1:**
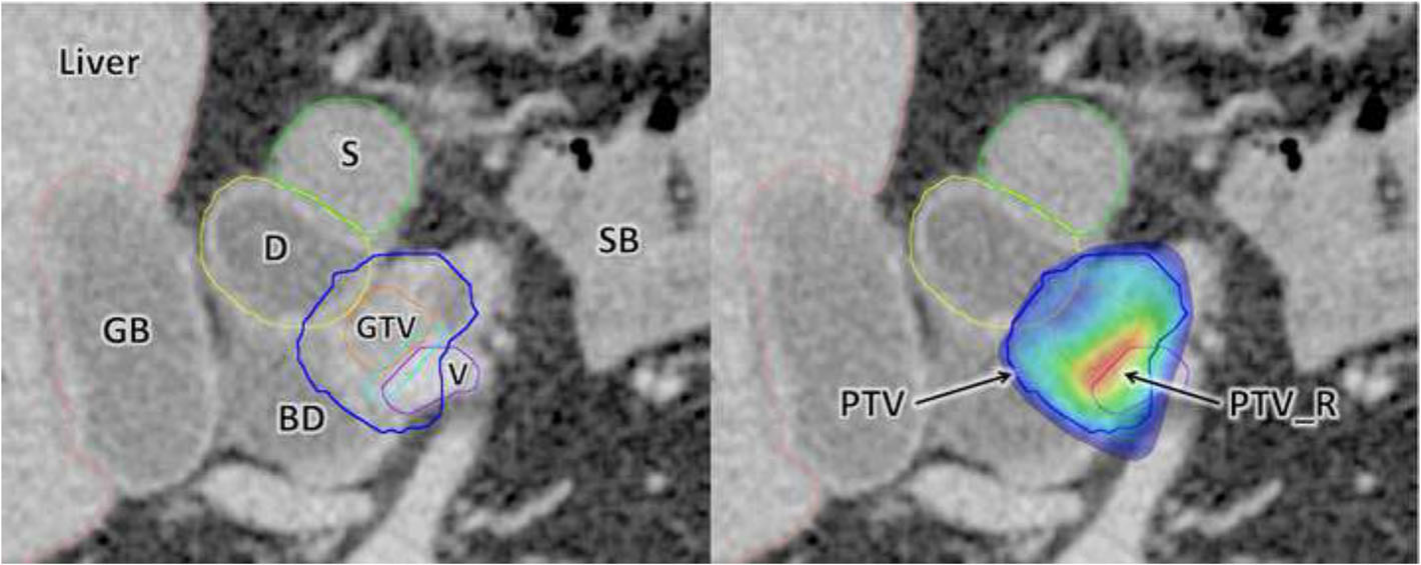
Axial contrast-enhanced CT of patient with borderline-resectable pancreatic cancer demonstrating SPARC radiotherapy planning. Left-hand image - delineated structures: (*clockwise from left*) GB = gall-bladder, D = duodenum, S = stomach, SB = small-bowel, V = vessel in contact with tumour, GTV = Gross Tumour Volume, BD = bile duct. Right-hand image - radiotherapy plan dose colourwash demonstrating dose levels delivered to PTV_R (*light blue contour*), PTV (*dark blue*) and PTV overlapping with duodenum

The concept of margin‐intensive therapy aims to further improve local control without increasing toxicity, as the region of integrated boost tends not to overlap with the major dose-limiting organ at risk (duodenum) helping to make safe dose-escalation to this sub-volume possible. Normal-tissue dose-volume constraints retain priority but where there is overlap, only the dose to the overlapping region is constrained as necessary, enabling the non-overlapping portion of the target volume to receive the prescribed dose (see Fig. 1).

The optimal pre-operative therapy in pancreatic cancer has not been identified, and could involve chemotherapy, radiotherapy or both. The national multicentre study ESPAC‐5 F is currently open to recruitment in the UK with the primary aim to establish the feasibility of recruiting patients with BRPC to be randomised between two pre‐operative chemotherapy regimens (GEMCAP and FOLFIRINOX), pre‐operative CRT and standard of care (surgery followed by adjuvant chemotherapy) (38), therapy options which would then require definitive comparative evaluation in a subsequent larger study.

Two single-institution retrospective reviews report outcomes for the use of SBRT in patients with BRPC, both of which show high rates of subsequent successful surgical resection with clear margins [38, 39]. Rajagopalan et al. [38] report on 12 resected patients, of which 7 were deemed BRPC using.

MDACC criteria. Most patients received Gemcitabine-based systemic chemotherapy. The SBRT consisted of 24–36 Gy in 3 fractions, surgery took place at a median 3.3 months (range 1.5 – 6.6 months) and 11/12 patients had an R0 resection. Chuong et al. [39] treated 73 patients (of which 57 were deemed BRPC using NCCN criteria) with systemic Gemcitabine-based chemotherapy and 5-fraction SBRT. Median dose prescribed was 35 Gy to the tumour margin and no less than 25 Gy to the entire pancreatic tumour. 44/57 BRPC cases underwent exploratory resection and 31/32 resected cases had an R0 resection. No acute toxicity > grade 3 occurred and 5.3 % (4 patients with locally advanced inoperable disease) experienced late grade 3 toxicity (3 GI bleeding and 1 anorexia). With a median FU of 10.5 months (2.2‐25.9 months) the median OS was 16.4 months in all BRPC patients, with median OS of 19.3 months in resected patients. Disappointing overall survival rates in published series (median overall survival 5–20 months) are attributed to variable provision of optimal systemic treatment.

Evidence is growing for the importance of Radiation Therapy Quality Assurance (RTQA) for clinical trial interpretation as well as patient outcomes [40]. The SPARC trial incorporates a comprehensive prospective RTQA programme with a detailed radiotherapy protocol and atlas that was supported by a pre-trial workshop for collaborating clinicians, both of which have been shown to reduce variation in target volume definition [41–43]. A pre-trial test case of contouring and planning must be completed satisfactorily by recruiting centres and RTQA will continue with on-trial peer review prior to treatment supported by web-conferencing facilities.

The trial originally opened with eligibility criteria that excluded patients with suspicion of metastatic disease, those who may not be fit for surgical resection, and those who had received induction chemotherapy, which has become more common practice since the study was initially designed. Many patients presenting to our centre with BRPC were rendered ineligible for these reasons, and during the first year that the trial was open only one patient was successfully recruited. The primary endpoint of the study is the assessment of the safety and tolerability of the intervention, and therefore the patient eligibility inclusion and exclusion criteria for the study were revised to the current version published here, with the aim of improving patient recruitment.

### Exploratory immunological investigations

Preclinical evidence suggests radiotherapy generates an immunological response, which can contribute to the efficacy of treatment. Direct damage and local inflammation increases antigen presentation to stimulate adaptive immune responses, and radiotherapy delivered in larger fractions has been shown to particularly enhance this effect [28]. In the SPARC study a panel of immunological tests to examine innate and adaptive immune responses to pancreatic SBRT. These preliminary data will be explored for their utility as predictive bio‐markers in future studies.

## Conclusion

The non‐interventional phase of follow‐up after the last patient last visit at the end of study will permit an estimate of the efficacy of pre‐operative SBRT on overall survival and progression free survival to be observed. Once the MTD has been defined in this phase I trial, a phase II study will definitively evaluate efficacy end‐points. If positive, subsequent randomised phase III investigations will be required to assess superiority of this treatment strategy alone or integrated with pre-operative systemic treatment over surgery alone for this patient group.

## Abbreviations

AHPBA: American Hepato-Pancreato-Biliary Association
ALP: Alkaline phosphatase
AST: Aspartate transaminase
BED: Biologically effective dose
BRPC: Borderline resectable pancreatic cancer
CI: Confidence interval
CRT: Chemo-radiotherapy
CRUK: Cancer Research United Kingdom
CSM: University of Oxford Centre for Statistics in Medicine
CT: Computed tomography
CTAAC: Cancer Research UK Clinical Trials Award and Advisory Committee
DLT: Dose-limiting toxicity
ESPAC: European study group for pancreatic cancer
EQD_2_: Equivalent dose in 2-Gy fractions
FOLFIRINOX: Folinic acid-5-fluourouracil-irinotecan-oxaliplatin
FU: Follow-up
GEMCAP: Gemcitabine-capecitabine
HR: Hazard ratio
MDACC: MD Anderson Cancer Centre
MDT: Multi-disciplinary team
MRI: Magnetic resonance imaging
MTD: Maximum tolerated dose
NCCN: National comprehensive cancer network
OCTO: Oncology clinical trials office
OCTRU: Oxford clinical trials research unit
OIRO: CRUK/MRC Oxford Institute for Radiation Oncology
OS: Overall survival
PFS: Progression-free survival
PV: Portal vein
REC: Research ethics committee
RIOC: Radiotherapy and imaging trials oversight committee
RTQA: Radiation therapy quality assurance
SBRT: Stereotactic body radiation therapy
SMA: Superior mesenteric artery
SMV: Superior mesenteric vein
SSAT: Society for surgery of the alimentary tract
SSO: Society of surgical oncology
